# A comparison of the sociodemographic, medical, and psychosocial characteristics of adolescents and young adults diagnosed with cancer recruited in-person and online: A Canadian cross-sectional survey

**DOI:** 10.1177/20552076231205278

**Published:** 2023-10-25

**Authors:** Jacqueline L Bender, Rukayyah Akinnibosun, Natasha Puri, Norma D’Agostino, Emily K Drake, Argerie Tsimicalis, A Fuchsia Howard, Sheila N Garland, Karine Chalifour, Abha A Gupta

**Affiliations:** 1 Cancer Rehabilitation and Survivorship, Department of Supportive Care, Princess Margaret Cancer Centre, University Health Network, Toronto, ON, Canada; 2 Dalla Lana School of Public Health, 7938 University of Toronto, Toronto, ON, Canada; 3 Institute of Health Policy, Management and Evaluation, 7938 University of Toronto, Toronto, ON, Canada; 4 Department of Supportive Care, Princess Margaret Cancer Centre, University Health Network, Toronto, ON, Canada; 5 Faculty of Health, Dalhousie University, Halifax, NS, Canada; 6 Ingram School of Nursing, Faculty of Medicine and Health Sciences, 5620 McGill University, Montreal, QC, Canada; 7 School of Nursing, 8166 The University of British Columbia, Vancouver, BC, Canada; 8 Department of Psychology, Memorial University, St John's, NL, Canada; 9 Young Adult Cancer Canada, St John's, NL, Canada; 10 Division of Medical Oncology and Hematology, Princess Margaret Cancer Centre, University Health Network, Toronto, Ontario, Canada; 11 Department of Pediatrics, University of Toronto, Toronto, Ontario, Canada

**Keywords:** Internet, social media, recruitment, cancer, adolescents and young adults

## Abstract

**Introduction:**

Adolescents and young adults diagnosed with cancer (AYAs) are under-represented in research. The Internet and social media could increase the reach of recruitment efforts but may impact sample characteristics. This study evaluated the characteristics of AYAs recruited in-person at an urban hospital versus the Internet in terms of their sociodemographic and medical characteristics, and psychosocial wellbeing, and offers recommendation for increasing the inclusivity and representativeness of research samples.

**Methods:**

Participant data from a cross-sectional survey of AYAs in Canada were evaluated. In-person hospital recruitment used a registry to identify patients attending ambulatory clinics. Internet recruitment included notices on hospital, team members’, and community partners’ social media channels, and email newsletters. Independent sample *t*-tests and Chi-squared tests were used to identify differences in participant sociodemographic, medical, and psychosocial characteristics based on recruitment source.

**Results:**

Of 436 participants, 217 (49.8%) were recruited in-person and 219 (50.2%) online. Online participants were more likely: to be white (*p* < .001), women (*p* < .001), and Canadian-born (*p* < .001); to speak English at home (*p* < .001), live alone (*p* = .001) and live in rural settings (*p* = .014); and to be farther from diagnosis (*p* = .023), diagnosed with breast cancer (*p* < .001), and cancer free (*p* < .001) compared to the hospital sample. Online participants also reported higher anxiety, depression, and loneliness (*p* < .001), and lower social support (p < .001), self-efficacy for coping with cancer (*p* < .001), and life satisfaction (*p* = .006).

**Conclusions:**

Online recruitment yielded a more geographically diverse but less sociodemographically diverse sample of AYAs who were farther from diagnosis and had poorer psychosocial wellbeing than in-person recruitment at an urban hospital. Future research efforts should consider partnering with under-represented communities and using targeted and stratified online and in-person recruitment strategies to achieve an inclusive and representative sample of AYAs.

## Introduction

Adolescents and young adults diagnosed with cancer (AYAs) experience unique challenges because of the timing of their diagnosis and treatment within the context of their lives.^1–4^ These challenges include high levels of symptom burden and treatment-related complications, interrupted education, careers and relationships, and major financial hardship.^5–8^ Yet, AYAs have been under-represented in research^4^ and have the lowest rate of clinical trial enrollment compared to all other age groups,^9–11^ rendering many of these impacts poorly understood. This may be due in part to recruitment challenges.^12–14^

Documented barriers to the inclusion of AYAs in research include limitations arising from traditional hospital-based methods of recruitment (e.g. in-person contact during healthcare appointments, patient identification through hospital EMRs, postal mail or telephone follow-up).^9,13,14^ Notably, fewer AYAs are seen in medical facilities compared to older age groups given the lower incidence of cancer in AYAs.^14^ Follow-up care of AYAs is dispersed across pediatric and adult primary care, tertiary care, and community settings.^14^ AYAs are a geographically dispersed population with fluctuating addresses as they often relocate for education, employment, and relationships.^14^ AYAs are also less likely to attend follow-up appointments due to prioritization of education, career, and family obligations, as well as the cost of accessing care.^15^

Internet-based recruitment could overcome many of the limitations of hospital-based recruitment and has been shown to be effective in recruiting AYAs.^16^ Social media recruitment offers several advantages, including access to a larger and potentially more diverse population, as well as smaller, hard-to-reach groups.^17^ Social media recruitment can also be more economical than offline recruitment by reducing the time, effort, and cost.^16^ For example, a Dutch study of fertility in young woman (18–45 years of age) reported that online recruitment (e.g. advertisements on websites and social media) yielded 82.4% of participants compared to 10.4% who were recruited offline (e.g. press releases, posters, and flyers), and was less costly.^14^

Despite potential advantages, less is known about the impact of Internet-based recruitment on the characteristics of the research sample. Different recruitment approaches may lead to over or under-representation of the target population that could bias findings and limit generalizability if unknown. For example, Benedict et al. compared online (e.g. social media) and offline (e.g. hospital-based EMR identification and postal letter and phone contact) approaches to recruit a U.S.-based sample of female AYA cancer survivors and found group differences in the characteristics and patient-reported outcomes of participants based on recruitment source.^13^ Specifically, participants recruited from social media were more likely to be white and reported higher levels of reproductive concerns and negative mood compared to participants recruited from a hospital.^13^

Research is needed to better understand the impact of recruitment source in a broader sample of AYAs, on a broader set of patient-reported outcomes, and in a different context. Thus, this study aimed to evaluate differences in the characteristics of Canadian AYAs recruited in-person at an urban hospital versus through the Internet in terms of their sociodemographic and medical characteristics, as well as their psychosocial wellbeing. This information is critical to help researchers and other stakeholders understand how recruitment approaches influence who is and who is not represented in a research study. We conclude with recommendations on how to increase the inclusivity and representativeness of research samples.

## Methods

### Study design

Data were obtained from the AYA Connect 4 Health Study—a sequential, explanatory, mixed-methods study^18^ of the peer support needs and digital peer navigation preferences of AYAs in Canada. Participants in the AYA Connect 4 Health Study completed a one-time paper or electronic cross-sectional survey and a subgroup participated in one of four in-person focus groups followed by an in-person co-design workshop. Ethical approval was obtained by the University Health Network (UHN) Research Ethics Board. This paper reports on the characteristics of individuals who participated in the cross-sectional survey, following the guidelines for Strengthening the Reporting of Observational Studies in Epidemiology (STROBE).^19^

### Participants

Individuals were eligible to participate in the survey if they were diagnosed with cancer between the ages of 15 and 39 years, able to read and speak English, and were receiving treatment for cancer or within 10 years of treatment completion.

### Recruitment settings

The survey was distributed in-person at the Princess Margaret Cancer Center (PM), as well as online from September 2018 to April 2019, one year prior to the COVID-19 pandemic. The PM is a tertiary, university-affiliated, teaching hospital located in the urban core of Toronto in Ontario, Canada. The PM has an AYA Program that provides specialized support to younger people (<40 years old) living with cancer. Patients who participate in the PM AYA Program meet one-on-one with an AYA Clinical Nurse Specialist. All the AYA resources and events are promoted online through the AYA PM social media channels and monthly e-newsletters. Survey participants were offered the opportunity to be entered into a draw to win one of three CAD $100 Amazon gift cards upon completion of the survey.

#### Hospital-based recruitment

At the hospital, AYAs were recruited using a weekly AYA report that identified all patients less than 40 years of age who had an appointment at a disease specific ambulatory clinic, including those diagnosed with: leukemia, lymphoma, breast cancer, genitourinary cancer, central nervous system cancer, head/neck cancer, sarcoma, gastrointestinal cancer, and gynecological cancer. Patients listed in the weekly AYA report were sequentially approached in clinic, informed about the survey, asked to confirm their eligibility, and if agreeable, given a paper copy of the survey that included a consent form which they were asked to read and sign indicating their agreement to participate before proceeding with the survey. Patients completed the survey in clinic and submitted it to a research team member or clinic administrator prior to departure or at their next clinic appointment if they required additional time. No passive recruitment strategies (e.g. study fliers or posters) were used. Our target sample for hospital-based recruitment was 200 to acquire sufficient power for multivariable analyses.

#### Internet-based recruitment

Online, AYAs were recruited using social media and email lists following a privacy-by-design recruitment framework developed by the lead author.^17^ REB approved recruitment notices included: a study description, varying images of young adults of different ages, genders and ethnicities, a disclaimer regarding the privacy limitations of social media, study team contact information, relevant hashtags, and a link to an online version of the survey and consent form. The recruitment notices were posted by the hospital's AYA Program on their social media channels every 1 to 2 weeks, including nine times on Twitter, six times on Facebook, and twice on Instagram (including Instagram Stories). In addition, we purchased three Boost Post ads to increase the reach of the recruitment notices to Facebook and Instagram users who were within our target demographic (e.g. 15–39), living in Canada, and who expressed an interest in, or liked pages related to cancer organizations. The three Boost Post ads each ran for 7 days on Facebook and Instagram approximately 4 weeks apart and were implemented when the online survey response rate started to decrease. The first Boosted recruitment post targeted males, the second targeted females, and the third targeted a general young adult population. We posted more on Twitter and Facebook than Instagram because the project team (which included community partners Young Adult Cancer Canada (YACC), and Adolescent and Young Adult Cancer Societal Movement (#AYACSM) Twitter community) were more active and had a larger network of followers on those social media platforms at that time. The team endorsed and promoted the recruitment notices that we posted on our hospital's AYA Program Twitter, Facebook, and Instagram accounts by sharing the posts and encouraging the AYA cancer community to re-share the posts. We provided the team with a social media strategy that included suggested social media messages and hashtags to accompany the re-sharing of recruitment posts, optimal times to re-share on Twitter, Facebook, and Instagram for maximal reach, and social media influencers in AYA oncology with whom to share the posts for further promotion. Lastly, we included the recruitment notice on the hospital's AYA Program e-newsletter that was distributed to AYA patients who had registered to receive it. Our target sample for Internet-based recruitment was 200 to facilitate comparison with the hospital sample.

### Survey

The self-report survey contained four sections: (a) cancer specific background information, (b) preferences for peer support from other AYAs living with cancer, (c) overall health and wellbeing, and (d) sociodemographic information. A full copy of the survey has been published elsewhere.^18^ This paper reports on responses to questions in sections A, C and D.

Sociodemographic information included: age at the time of study, gender, sexual orientation, race/ethnicity, languages spoken at home, highest level of education, personal income, current school or employment status, household living arrangement, geographic setting, and country of birth. Medical information included: age at diagnosis, diagnosis date, tumor type and stage, treatment status, treatments received, remission status. Psychosocial wellbeing was assessed using standardized patient-reported-outcome (PROs) measures of anxiety, depression, loneliness, social support, and coping behavior that have been shown to be valid in cancer patients and have been used in research with AYAs. Anxiety was measured using the Generalized Anxiety Disorder Scale (GAD-7); clinical significance is ≥ 8.^20^ Depression was measured using the Patient Health Questionnaire (PHQ-9); clinical significance is ≥10.^21^ Loneliness was measured using the UCLA Loneliness Scale (version 3).^22^ Social support was measured using the Social Provisions Scale (SPS-10).^23^ Self-efficacy for coping was measured using the Cancer Behavior Inventory (CBI; version 3).^24^ Lastly, life satisfaction was measured using the life satisfaction scale used in the Canadian Community Health Survey.^25^

### Analysis

Statistical analyses were conducted using SPSS version 25. Means and standard deviations were calculated for continuous variables and proportions for categorical variables. Independent sample *t*-tests and Chi-squared tests were conducted to assess differences in sociodemographic and medical characteristics and PROs by recruitment source. Fisher's exact tests were conducted for variables with cell counts less than 5. Group differences of *p* < .05 were considered significant.

## Results

### Participant characteristics

Four hundred and thirty-six participants completed the survey, of whom 217 (49.8%) were recruited in-person and 219 (50.2%) were recruited online. On average, participants were 31 years of age and 3.3 years post diagnosis (Table 1). Most were diagnosed with breast cancer (*n* = 91, 21.7%) or Hodgkin's lymphoma (*n* = 47, 11.2%). About two-thirds of the participants identified as a woman (*n* = 218, 65.1%), heterosexual (*n* = 286, 64.6%), and white (*n* = 212, 63.1%), and had completed at least some university (*n* = 216, 63.1%). Most of the participants were urban or suburban residents (*n* = 292, 86.9%) and from of the province of Ontario (*n* = 274, 81.3%).

**Table 1.** Sociodemographic differences.

*Sociodemographic characteristics*					
	Total sample*N*Mean (SD)Or *n* (%)	In-person, hospital sample*N*Mean (SD)Or *n* (%)	Online sample*N*Mean (SD)Or *n* (%)	*t-Test, Chi squared or Fisher's exact	*p*-Value*
**Age**	419	215	204	5.56	<.001
	31.27 (6.4)	29.7 (6.4)	32.9 (5.8)		
**Gender**	335	203	134	63.97	<.001
Man	117 (34.9)	105 (51.7)	12 (9.1)		
Woman	218 (65.1)	98 (48.3)	120 (90.9)		
Other	0 (0)	0 (0)	0 (0)		
**Race/ethnicity**	336	202	134	52.63	<.001
Indigenous	4 (1.2)	1 (0.5)	3 (2.2)		
White	202 (60.1)	95 (47.0)	107 (79.9)		
Afro-Caribbean	18 (5.4)	16 (7.9)	2 (1.5)		
Asian	45 (13.4)	31 (15.3)	14 (10.4)		
South Asian	20 (6.0)	20 (9.9)	0 (0)		
Middle Eastern	20 (6.0)	18 (8.9)	2 (1.5)		
Other	27 (8.0)	21 (10.4)	6 (4.5)		
**Born in Canada**	337	203	134	17.10	<.001
Yes	251 (74.5)	135 (66.5)	116 (86.6)		
No	86 (25.5)	68 (33.5)	18 (13.4)		
**Language spoken at home**	321	187	134	13.44	<.001
English	282 (87.9)	161 (86.1)	121 (90.3)		
French	8 (2.5)	1 (0.5)	7 (5.2)		
Other	31 (9.7)	25 (13.4)	6 (4.5)		
**Household arrangement**	337	203	134	6.05	.014
Live alone	42 (12.5)	18 (8.9)	24 (17.9)		
Live with others	295 (87.5)	185 (91.1)	110 (82.1)		
**Location setting**	336	202	134	6.06	.014
Urban/suburban (city)	292 (86.9)	183 (90.6)	109 (81.3)		
Town/rural (country)	44 (13.1)	19 (9.4)	25 (18.7)		
**Province or territory where you currently live**	335	203	132	–	–
Newfoundland and Labrador	6 (1.8)	0 (0)	6 (4.5)		
Prince Edward Island	0 (0)	0 (0)	0 (0)		
Nova Scotia	6 (1.8)	0 (0)	6 (4.5)		
New Brunswick	0 (0)	0 (0)	0 (0)		
Quebec	11 (3.3)	0 (0)	11 (8.3)		
Ontario	274 (81.8)	202 (99.5)	72 (54.5)		
Manitoba	14 (4.2)	13 (9.8)	1 (0.5)		
Saskatchewan	1 (0.3)	0 (0)	1 (0.8)		
Alberta	13 (3.9)	0 (0)	13 (9.8)		
British Columbia	9 (2.7)	0 (0)	9 (6.8)		
Yukon	0 (0)	0 (0)	0 (0)		
Northwest Territories	1 (0.3)	0 (0)	1 (0.8)		
Nunavut	0 (0)	0 (0)	0 (0)		
**Education**	340	203	137	5.92	.055
Less than secondary school	8 (2.4)	8 (3.9)	0 (0)		
Secondary school	88 (25.9)	52 (25.6)	36 (26.3)		
Post-secondary degree	244 (71.8)	143 (70.4)	101 (73.7)		
**Income**	259	150	109	0.313	.855
Less than $40,000	123 (47.5)	71 (47.3)	52 (47.7)		
$40,000–$80,000	87 (33.6)	49 (32.7)	38 (34.9)		
More than $80,000	49 (18.9)	30 (20.0)	19 (17.4)		
**Sexual orientation**	327	198	129	1.76	.627
Heterosexual	286 (85.4)	176 (53.8)	110 (33.6)		
Homosexual	15 (4.6)	9 (2.8)	6 (1.8)		
Bisexual	21 (6.3)	10 (3.1)	11 (3.4)		
Other	5 (1.5)	3 (0.9)	2 (0.6)		

**Table 2.** Medical differences.

*Medical characteristics*					
	Total sample*N*Mean (SD)Or *n* (%)	In-person, hospital sample*N*Mean (SD)Or *n* (%)	Online sample*N*Mean (SD)Or *n* (%)	*t*-Test or Chi squared or Fisher's exact	*p*-Value
**Time since diagnosis (years)**	415	212	203	2.28	.023
	3.30 (3.79)	2.89 (3.75)	3.73 (3.79)		
**Cancer type**	421	217	204	78.52	<.001
Breast	91 (21.7)	19 (8.8)	72 (35.3)		
Gynecological (cervical, ovarian, uterine)	27 (6.4)	13 (6.0)	14 (6.9)		
GI, colorectal or liver	31 (7.9)	13 (6.0)	18 (8.8)		
Hodgkin's lymphoma	47 (12.0)	25 (11.6)	22 (10.8)		
Non-Hodgkin's lymphoma	25 (6.0)	9 (4.2)	16 (7.8)		
Leukemia	36 (8.6)	23 (5.9)	13 (3.3)		
Testicular	41 (9.8)	39 (18.1)	2 (1.0)		
Thyroid	32 (7.6)	20 (9.3)	12 (5.9)		
Sarcomas	35 (8.4)	25 (11.6)	10 (4.9)		
Other	54 (12.9)	29 (13.5)	25(12.3)		
**Cancer stage at first diagnosis**	300	131	169	6.69	.082
Stage 1	80 (26.7)	43 (32.8)	37 (21.9)		
Stage 2	91 30.3)	38 (29.0)	53 (31.4)		
Stage 3	87 (29.0)	30 (22.9)	57 (33.7)		
Stage 4	42 (14.0)	20 (15.3)	22 (13.0)		
**Currently living with metastatic cancer**	413	208	205	0.53	.465
No	345 (83.5)	171 (82.2)	174 (84.9)		
Yes	68 (16.5)	37 (17.8)	31 (15.1)		
**Cancer free**	353	170	183	12.55	<.001
No	117 (33.1)	72 (42.4)	45 (24.6)		
Yes	236 (66.9)	98 (57.6)	138 (75.4)		

**Table 3.** Psychosocial wellbeing differences.

	Total sample*N*Mean (SD)Or *n* (%)	In-person, hospital sample*N*Mean (SD)Or *n* (%)	Online sample*N*Mean (SD)Or *n* (%)	*t*-Test or Chi squared or Fisher's exact	*p*-Value
**Anxiety**	358	205	153	4.34	<.001
(GAD-7 Summary Score)	5.85 (5.06)	4.87 (1.67)	7.16 (5.28)		
**Levels of anxiety**	358	205	153	16.13	<.001
(GAD-7 cut offs)					
Minimal (0–4)	163 (45.5)	110 (53.7)	53 (34.6)		
Mild (5–9)	119 (33.2)	64 (31.2)	55 (35.9)		
Moderate (10–14)	47 (13.1)	20 (9.8)	27 (17.6)		
Severe (15–21)	29 (8.1)	11 (5.4)	18 (11.8)		
**Depression**	354	204	150	4.59	.001
(PHQ-9 Summary Score)	6.41 (5.66)	5.25 (5.26)	7.97 (5.83)		
**Levels of depression**	354	204	150	20.40	<.001
(PHQ-9 cut offs)					
Minimal (0–4)	170 (48.0)	118 (57.8)	52 (34.7)		
Mild (5–9)	98 (27.7)	49 (24.0)	49 (32.7)		
Moderate (10–14)	49 (13.8)	23 (11.3)	26 (17.3)		
Moderately severe (15–19)	27 (7.6)	10 (4.9)	17 (11.3)		
Severe (20–27)	10 (2.8)	4 (2.0)	6 (4)		
**Loneliness**	343	198	145	6.66	<.001
(UCLA Loneliness Score)	15.82 (14.78)	11.54 (13.13)	21.68 (14.93)		
**Social support**	349	199	150	-4.02	<.001
(SPS Summary Score)	35.02 (4.97)	35.92 (4.61)	33.81 (5.17)		
**Coping behaviors inventory**	301	172	129	-5.21	<.001
(CBI Summary Score)	135.43 (27.51)	142.3 (26.72)	126.28 (25.94)		
**Life satisfaction**	357	202	155	-2.77	.006
	6.76 (1.99)	7.01 (2.11)	6.43 (1.78)		

### Sociodemographic differences

Participants recruited online were significantly more likely to be older (*p* < .001), white (*p* < .001), women (*p* < .001), and Canadian-born (*p* < .001), and to speak English at home (*p* < .001) compared to the hospital sample (Table 1). They were also more geographically dispersed across the country and more likely to be living alone (*p* = .014) and residing in small towns or rural settings (*p* = .014).

### Medical differences

Participants recruited online were more likely to be farther from diagnosis (*p* = .023), diagnosed with breast cancer (*p* < .001), and cancer free (*p* < .001) in comparison to the hospital sample (Table 2).

### Psychosocial wellbeing differences

Participants recruited online reported significantly higher levels of anxiety (*p* < .001), depression (*p* < .001), and loneliness (*p* < .001), and lower levels of perceived social support (*p* < .001), and self-efficacy for coping with cancer (*p* < .001) compared to the hospital sample. They were also more likely to report lower life satisfaction (*p* < .006) (Table 3).

## Discussion

### Summary

Achieving a representative sample of the population and minimizing selection bias is essential for the generalizability of survey research.^26^ Given the challenges recruiting AYAs for research studies and the potential reach of online methods, this study evaluated the impact of in-person hospital-based recruitment compared to Internet-based recruitment on the characteristics of the study sample. While online recruitment yielded a more geographically diverse sample with participants from different provinces and territories across Canada, online participants were significantly less sociodemographically diverse than those recruited at the hospital. Participants recruited online also reported significantly poorer psychosocial wellbeing and less life satisfaction than those recruited at the hospital. These findings align with the demographic, medical, and psychosocial differences observed by Benedict et al.,^13^ in their comparison of urban hospital versus social media recruitment of young women who participated in a fertility study. To our knowledge, this is the first study to investigate the impact of hospital versus online recruitment on the sociodemographic, medical, and psychosocial characteristics of a broad sample of AYAs that included males.

### Why were fewer male AYAs recruited online?

Under-representation of men in web-based health research is not uncommon. A systematic review of social media health behavior interventions reported that 83.3% of study participants were female.^27^ Similarly, in a national survey of quality of life in AYA cancer survivors that used social media recruitment methods, 82.2% of participants were female.^28^ Lower enrollment of male AYAs via social media may have occurred because fewer males compared to females use social media to obtain cancer-related support.^29^ In a previous study of cancer patients recruited from the same hospital that included AYAs, we demonstrated that females were more than twice as likely to use social media for cancer-related support compared to males.^29^ Females have historically been more avid social media users than males, although the sex gap is decreasing.^30^ Females are also more likely to seek support for health-related concerns than males.^29^ Gender differences in support seeking, which may reflect gendered coping styles influenced by social norms,^31^ could have prevented men from participating in the study. For example, a study of men's views on cancer rehabilitation revealed that ideals associated with traditional notions of masculinity were barriers to their participation. These included fear of losing control (e.g. a fear of reduced manliness, sympathy and dependency, confrontation with death) and striving for normality (e.g. a desire for autonomy and purpose, solidarity, and to forget and move on).^32^

In addition, the social media strategy may not have reached as many male AYAs who were on social media. We used Facebook, Twitter, and Instagram to promote the study and purchased Facebook ads to target and boost the reach of our recruitment posts to males on Facebook and Instagram. However, Facebook and Instagram are used by about 10% to 15% more females than males.^33^ More Facebook ads targeting young males, as well as promotion via platforms that attract more males such as Reddit^33^could increase the delivery of recruitment posts to young males. Further tailoring of the recruitment message, such as the inclusion of images of young males in all posts may help to increase the perceived relevance to males as well. However, tailoring alone may be insufficient to boost enrollment of young males in research. Ryan et al.^34^ reported that social media posts with images of vibrant healthy males exercising generated the most clicks but did not increase participation of males in a physical activity trial. Only encouraging females to invite their male friends to join the study boosted participation of males.

### Why were fewer racially and ethnically diverse AYAs recruited online?

Under-representation of racial and ethnic minorities in research is a long-standing concern reflective of a legacy of neglect and mistreatment in research.^35,36^ Encouragingly, both the in-person and online samples were more racially diverse than a previous study involving cancer patients from the same hospital^29^ and a cross-Canada study of AYAs recruited through social media.^37^ Importantly, although our online sample was considerably less racially diverse than the hospital sample (e.g. 21.1% online vs. 53% in hospital), it was representative of the racial diversity of the Canadian population. In addition, the hospital sample was representative of the racial diversity of Toronto, the city in which the hospital was located. Canada is a racially and ethnically diverse county, with a visible minority population of 7.6 million.^38^ In 2016, 22.3% of Canadians identified as a visible minority, of whom, three in 10 were born in Canada.^38^ A higher proportion of Canadians who identify as racialized live in urban metropolitan areas, with the city of Toronto being one of the most culturally diverse cities in the country, and where 52% of residents identify as racialized.^39^

In addition to geographic considerations, the social media strategy may not have reached as many racially diverse AYAs on social media. Our social media recruitment efforts relied on the online communities and social networks of a cancer center, a cancer support agency, and a AYA Twitter community. Although equal proportions of racialized and non-racialized people use social media,^33^ English language online cancer communities have historically been populated by people who identify as white.^40–42^ Identified barriers to the participation of African and Asian American cancer patients in online cancer communities include ethnic differences in attitudes toward support seeking, the need for culturally safe support, and previous experiences of discrimination in healthcare encounters.^40,41^ Strategies to increase the participation of racially and ethnically diverse cancer patients in research studies include ensuring representation of under-represented communities on the project team, partnership with under-represented communities, community-based recruitment, and culturally tailored recruitment strategies.^43–45^ While our team included members who were diverse in gender, race, and ethnicity, most identified as white. Further, while we tailored our recruitment materials to reach diverse AYAs, we did not engage in community-based recruitment with underrepresented communities.

### Why was the online sample more distressed?

The online sample was considerably more distressed than the hospital sample, with higher proportions of AYAs reporting clinically significant levels of anxiety and depression, and lower coping self-efficacy. AYAs recruited online may be more distressed and may turn to cancer online communities as a coping strategy.^46^ In previous work, we demonstrated an association between distress and use of social media for cancer-related support in a sample of cancer patients (including AYAs) recruited from the same hospital as this study.^29^ Likewise, others have shown that cancer survivors who join cancer online communities are more distressed that those who do not.^47^ The medical characteristics and care setting of the online sample likely contributed to their higher levels of distress. A greater proportion of the online sample was farther from diagnosis and cancer free. This may mean that the social media strategy was more effective in recruiting AYA cancer survivors. Studies have documented high levels of distress^37^ and high levels of unmet supportive care needs^48^ in AYA cancer survivors several years after treatment. However, the online sample likely no longer had access to the same level of support from their health care team because of less frequent follow-up appointments. Further, a greater proportion of the online sample lived in rural settings and may have had unaddressed psychosocial concerns because of the lack of specialized AYA survivorship care in rural Canada.^49^

The online sample also reported higher levels of loneliness and lower levels of perceived social support than the hospital sample, as well as lower life satisfaction. Loneliness is a major issue among AYAs,^50^ who report limited opportunities to interact with other AYAs and encounter significant challenges forming new social relationships.^51^ Studies have shown that cancer survivors often turn to cancer online communities after treatment to find and obtain support from cancer peers.^52^ Hence, our social media recruitment strategy may have succeeded in reaching a subgroup of AYA cancer survivors with clinically significant levels of distress and low perceived social support who were using online cancer communities to seek support from cancer peers. Researchers should consider using social media interventions to reach and address the needs of this subgroup of AYA cancer survivors who are distressed and lacking social support. Coping self-efficacy may be a good intervention target given that many AYAs lack the life experience and skills to effectively cope with cancer. Patients who have higher coping self-efficacy are better able to manage the challenges associated with cancer and report less depression and anxiety, and a higher quality of life.^24^

## Recommendations for increasing the inclusivity of research samples

The utility of a recruitment strategy is dependent on its ability to recruit a representative sample of a target population. Ensuring the inclusion of individuals who are typically under-represented in research is critical to advance science, ensure the health and wellbeing of all members of society, and achieve health equity. We offer the following recommendations for increasing the inclusivity and representativeness of research samples.

### Partner with underrepresented communities

Community-based recruitment and culturally relevant and targeted recruitment strategies have been shown to enhance the inclusivity of research samples.^40–42^ Critical to the success of community-academic partnerships is the establishment of long-term partnerships with communities based on trust, equitable relationships, community-based data collection approaches, and data governance. In another project, the lead author has a long-standing partnership with a Black men's health organization which has facilitated the meaningful engagement of Black peer navigators and patient participants for a clinical trial of a digital peer navigation program^53^ that she developed.^54^ This has involved community-led recruitment at community events (e.g. prostate awareness nights and men's groups at churches) and tailored marketing materials featuring Black men.

### Use targeted, stratified sampling

Steele et al.^55^ used targeted convenience sampling to recruit self-identified sexual and gender minority people and heterosexual, cisgendered adult women for a cross-sectional Internet survey. This involved stratified sampling to ensure they met target numbers for nine mutually exclusive groups that had different intersections of sociodemographic factors and oversampling racialized and low-income women to ensure adequate representation of vulnerable groups. The group definitions were developed in collaboration with a community advisory group and consecutive participants were enrolled until the target samples size for each group was reached. Effort was placed on recruiting persons from vulnerable, under-represented groups first.

### Use multiple in-person and online recruitment strategies

The results from this research study suggest that both online and in-person recruitment strategies may be required to produce a representative sample of AYAs who are diverse in sociodemographic, medical and psychosocial characteristics. We also recommend offering a pen and paper version of the survey to remove barriers to participation for those who may not have access to computers, the Internet, or social media. Likewise, Steele et al.^55^ supplemented their digital recruitment methods by mailing hard copies of study flyers to key health and social services agencies and visiting key community agencies in person to promote the project and facilitate recruitment of individuals belonging to their target identity groups.

### Target influential social network members

As shown by Ryan et al.,^34^ the most effective strategy to increase the participation of males in their research study was encouraging females to invite their male friends to join. For AYAs, this could also include explicitly targeting their family caregivers (e.g. parents or spouses) or friends, including “bring a buddy” recruitment campaigns. In addition, social media strategies could benefit from partnering with cancer organizations that represent under-represented communities and that have a social media presence.

## Limitations

The study has certain limitations. The convenience sample may have introduced selection bias towards AYAs who are interested in peer support and those who had access to social media, for the online recruitment. The degree of selection bias is likely higher among those recruited online versus those approached in clinics, as there was an effort made to approach every eligible hospital patient during the study period. The cross-sectional survey likely introduced recall bias. Notably, a considerable proportion of respondents reported that they did not know aspects of their medical information, resulting in smaller sample sizes for those variables. We did not collect data on the extent to which the social media strategy was implemented by the study team or endorsed or shared by the AYA community; hence we cannot comment on the reach and effectiveness of the social media strategy. It is possible that some of the participants recruited in-person from hospital clinics viewed the online recruitment notices, and vice versa. While this would be a limitation in terms of identifying the most effective recruitment method, that was not our intent. Rather, our goal was to compare the characteristics of those recruited in-person versus online. For those who may have been exposed to both in-person and online recruitment methods, whichever method effectively recruited them was what we documented. Given the potential for deception in web-based research^48^ the online sample may have included fraudulent responses. However, we implemented well-known safeguards to mitigate this risk including using a lottery for compensation instead of gift cards for each completed survey and removed incomplete surveys.^48^ Lastly, the hospital-based sample was recruited from a cancer center with a specialized survivorship program for AYAs located in a culturally diverse city, recruitment from less resourced hospitals located in less ethnically diverse geographic areas may yield different findings.

## Conclusion

Internet and social media-based recruitment of AYAs yielded a more geographically diverse but less sociodemographically diverse sample of AYAs who were farther from diagnosis and had poorer psychosocial wellbeing than in-person, urban hospital-based recruitment. Future research efforts should consider partnering with under-represented communities and using targeted and stratified online and in-person recruitment strategies to achieve an inclusive and representative sample of AYAs.

## Notes

**Correction (March 2025):** Article updated; for further details please see https://doi.org/10.1177/20552076251325521.
