# Erratum to “A comparison of the sociodemographic, medical, and psychosocial characteristics of adolescents and young adults diagnosed with cancer recruited in-person and online: A Canadian cross-sectional survey”

**DOI:** 10.1177/20552076251325521

**Published:** 2025-03-25

Bender JL, Akinnibosun R, Puri N, et al. A comparison of the sociodemographic, medical, and psychosocial characteristics of adolescents and young adults diagnosed with cancer recruited in-person and online: A Canadian cross-sectional survey. *Digit Health* 2023; 9. DOI: 10.1177/20552076231205278.

In the above-mentioned article, the affiliation of co-author, “Emily K Drake” was listed as “Department of Supportive Care, Princess Margaret Cancer Centre, University Health Network, Toronto, ON, Canada”. The following new affiliation “Faculty of Health, Dalhousie University, Halifax, NS, Canada” has been added leading to the renumbering of subsequent affiliations. Hence, the corrected affiliation of “Emily K Drake” should be read as:

^5^Faculty of Health, Dalhousie University, Halifax, NS, Canada

Additionally, “EKD runs her own consulting business” has been added to the “Declaration of conflicting interests” section. The revised statement reads as:

**Declaration of conflicting interests:** The authors declared no potential conflicts of interest with respect to the research, authorship, and/or publication of this article. EKD runs her own consulting business.

The article has been updated to reflect the corrections.

